# Therapeutic Potential of Green Synthesized Copper Nanoparticles Alone or Combined with Meglumine Antimoniate (Glucantime^®^) in Cutaneous Leishmaniasis

**DOI:** 10.3390/nano11040891

**Published:** 2021-03-31

**Authors:** Aishah E. Albalawi, Sobhy Abdel-Shafy, Amal Khudair Khalaf, Abdullah D. Alanazi, Parastoo Baharvand, Katrin Ebrahimi, Hossein Mahmoudvand

**Affiliations:** 1 Faculty of Science, University of Tabuk, Tabuk 47913, Saudi Arabia; ae.Albalawi@ut.edu.sa; 2 Department of Parasitology and Animal Diseases, Veterinary Research Division, National Research Centre, 33 Bohouth St., Dokki, Giza 12622, Egypt; aasobhy@yahoo.com; 3 Department of Microbiology, College of Medicine, University of Thiqar, Thiqar 0096442, Iraq; amalkhudair111@yahoo.com; 4 Department of Biological Science, Faculty of Science and Humanities, Shaqra University, P.O. Box 1040, Ad-Dawadimi 11911, Saudi Arabia; aalanazi@su.edu.sa; 5 Alghad International Colleges for Applied Medical Science, Tabuk 47913, Saudi Arabia; 6 Department of Community Medicine, Lorestan University of Medical Sciences, Khorramabad 68138-33946, Iran; dr.baharvand@gmail.com; 7 Department of Biology, Payame Noor University, Tehran 19569, Iran; ebr_k@yahoo.com; 8 Razi Herbal Medicines Research Center, Lorestan University of Medical Sciences, Khorramabad 68149-93165, Iran

**Keywords:** nanomedicine, amastigote, nitric oxide, cytotoxicity, cutaneous leishmaniasis, mice

## Abstract

Background: In recent years, the focus on nanotechnological methods in medicine, especially in the treatment of microbial infections, has increased rapidly. Aim: The present study aims to evaluate in vitro and in vivo antileishmanial effects of copper nanoparticles (CuNPs) green synthesized by *Capparis spinosa* fruit extract alone and combined with meglumine antimoniate (MA). Methods: CuNPs were green synthesized by *C. spinosa* methanolic extract. The in vitro antileishmanial activity of CuNPs (10–200 µg/mL) or MA alone (10–200 µg/mL), and various concentrations of MA (10–200 μg/mL) along with 20 μg/mL of CuNPs, was assessed against the *Leishmania major* (MRHO/IR/75/ER) amastigote forms and, then tested on cutaneous leishmaniasis induced in male BALB/c mice by *L. major.* Moreover, infectivity rate, nitric oxide (NO) production, and cytotoxic effects of CuNPs on J774-A1 cells were evaluated. Results: Scanning electron microscopy showed that the particle size of CuNPs was 17 to 41 nm. The results demonstrated that CuNPs, especially combined with MA, significantly (*p* < 0.001) inhibited the growth rate of *L. major* amastigotes and triggered the production of NO (*p* < 0.05) in a dose-dependent manner. CuNPs also had no significant cytotoxicity in J774 cells. The mean number of parasites was significantly (*p* < 0.05) reduced in the infected mice treated with CuNPs, especially combined with MA in a dose-dependent response. The mean diameter of the lesions decreased by 43 and 58 mm after the treatment with concentrations of 100 and 200 mg/mL of CuNPs, respectively. Conclusion: The findings of the present study demonstrated the high potency and synergistic effect of CuNPs alone and combined with MA in inhibiting the growth of amastigote forms of *L. major,* as well as recovery and improving cutaneous leishmaniasis (CL) induced by *L. major* in BALB/c mice. Additionally, supplementary studies, especially in clinical settings, are required.

## 1. Introduction

Cutaneous leishmaniasis (CL) is a vector-borne protozoan disease that is caused by the genus *Leishmania* and transmitted through the bite of female sandflies of *Phlebotomus* and *Lutzomyia* [1]. Based on the previous reports, CL-causing *Leishmania* are divided into two main categories: (i) Old World *Leishmania,* such as *L. major* and *L. tropica* (the most common species in the Middle East, Africa, and the Indian subcontinent); (ii) New World *Leishmania,* such as *L. amazonensis*, *L. mexicana*, *L. braziliensis*, etc. (which are prevalent in Middle and South America) [2]. Cutaneous leishmaniasis is described by a single or multiple long-lasting skin wounds and permanently disfiguring scars in the infected area [2,3].

Due to the unavailability of an effective vaccine and the appearance of drug resistance, efforts to treat and control CL are faced with serious challenges. Therefore, supporting and upgrading the present treatment agents, as well as discovering new and alternative agents, are considered to be important strategies for CL control and management [4]. The first-choice chemotherapy agents (meglumine antimoniate and sodium stibogluconate) and second-line drugs have some problems, such as the duration of therapy, adverse side effects, and emerging resistance in some species; these problems are limitations in CL treatment [5,6].

In recent years, attention to nanotechnological methods in medicine, especially for treating microbial infections, has been increasing markedly [7]. One of the most important applications of nanotechnology in medicine is to develop and improve drug delivery systems [8]. This field is based on the application of a number of drug-loaded nanocarrier systems, such as organic (chitosan, polymeric, etc.) and inorganic (metals, metal oxide, metal salts, etc.) nanoparticles, nanoemulsions, and nanostructured lipid carriers, etc. that facilitate targeted delivery, improve bioavailability, and minimize the toxicity of drugs to treat various infectious diseases [8].

Nowadays, nanoparticles are synthesized through various chemical and physical approaches. Among the available approaches, the “green synthesis” of nanoparticles using plant extracts, because of benefits such as cost-effectiveness, convenient preparation, and nontoxic constituents, along with adaptability with used medical and nutritional strategies, is considered to be one of the best methods for synthesizing nanoparticles [9].

Copper (Cu), one of the most helpful elements, has a wide range of pharmacological characteristics, including improving the immune system and having anti-inflammatory, antinociceptive, and antimicrobial properties [10]. In recent years, several studies have demonstrated that Cu nanoparticles (CuNPs), due to their high surface-to-volume ratio, are very reactive and merely interact with other particles, which results in various biological and therapeutic activities [10]. Currently, combination therapy is considered to be an effective plan to treat a wide range of microbial infections such as tuberculosis, malaria, AIDS, etc. [11]. Studies demonstrated that combination therapy has been progressively encouraged as a reliable approach to improve treatment efficacy and tolerance, decrease the treatment period and cost, and bound the emergence of drug resistance to treat leishmaniasis [12]. Based on what was said, the current research aims to assess the leishmanicidal activity of CuNP green synthesized by *Capparis spinosa* fruit methanolic extract alone and combined with MA in vitro and in vivo.

## 2. Materials and Methods

### 2.1. Copper Nanoparticles’ Green Synthesis

*C. spinosa* materials (fruits) were extracted and prepared through percolation techniques by means of 80% methanol for 3 days at room temperature. CuNPs were green synthesized based on the process explained elsewhere [13]. Succinctly, 75 mL of the extract mentioned earlier was added to 100 mL 0.01 M copper sulfate (CuSO4) solution (5H_2_O); it was then stirred at 60 °C for 24 h. Afterwards, it was centrifuged twice at 12,000 rpm for 15 min. The color change of the solution from green to yellow indicated the synthesis of nanoparticles in the solution. Finally, the obtained nanoparticles were dried at 60 °C for 120 min for further testing. The UV-visible spectrum, Fourier transform infrared spectroscopy (FTIR) analysis, and the specifications of the obtained nanoparticles, such as size and morphology, were previously recorded by a scanning electron microscope (Mira3, Brno, Czech Republic) with 15 kv, magnification of 10×, and resolution of 1 nm [13].

### 2.2. Parasite and Cell Culture

*L. major* (MRHO/IR/75/ER) promastigotes were cultured in RPMI 1640 (Sigma-Aldrich, Steinheim, Germany) improved with heat-inactivated fetal calf serum (FCS) (Sigma-Aldrich), streptomycin (100 μg/mL), and penicillin (200 IU/mL). J774-A1 murine macrophage cell line was cultured in Dulbecco’s modified Eagle’s medium (DMEM, Sigma-Aldrich) complemented with 10% FCS at 37 °C in 5% CO_2_.

### 2.3. In Vitro Antiamastigote Effects

The in vitro anti-intracellular amastigote activity of CuNPs alone and in combination with MA was accomplished based on the technique defined previously [14]. At first, J774-A1 (1 × 10^5^/mL) was placed in eight-chamber LabTek tissue-culture slides and incubated at 37 °C in 5% CO_2_ for 2 h in DMEM. In the next step, adherent macrophages were incubated with *L. major* promastigotes (10^6^/mL) in the stationary phase at the *Leishmania*/macrophage ratio of 10:1 and for one day. After removing the free promastigotes by washing with RPMI 1640 medium, the infected J774-A1 were exposed to some concentrations of CuNPs or MA (Glucantime, with 99% purity) alone (10–200 µg/mL) and several concentrations of MA (10–200 μg/mL) together with 20 μg/mL of CuNPs at 37 °C in 5% CO_2_ for 72 h. After fixing the slides with methanol and, then staining them with Giemsa, they were examined with a light microscope. Infected macrophages with no drug and the noninfected nontreated were measured as positive and negative controls, respectively. Anti-intracellular amastigote activity of CuNPs was determined by calculating the mean number of amastigotes in each macrophage after observing 100 J774-A1 cells compared with those in control groups. We calculated the 50% inhibitory concentrations (IC_50_ values) for all the studied groups by means of the Probit test using SPSS software 17.0 (SPSS Inc., Chicago, IL, USA). The examinations were carried out in triplicate.

### 2.4. Evaluating Inhibition of Infection in Macrophage Cells

In the present study, to examine the inhibitory effect of CuNPs against the promastigote invasion of macrophages, *L. major* promastigotes (10^6^/mL) were preincubated in CuNPs (10 and 20 µg/mL) alone and combined with MA for 2 h at 21 °C (the choice of these concentrations was based on the preliminary tests, which demonstrated no toxicity promastigote viability). In the next step, the washed promastigotes were exposed to J774 cells for 4 h. After fixing the slides with methanol and, then staining them with Giemsa, they were examined with a light microscope to assess the inhibition of infection through calculating 100 J774 cells [15].

### 2.5. Determining the Nitric Oxide (NO) Production

The Griess reaction (A and B, Sigma-Aldrich) for nitrites was used to evaluate the NO release in the supernatants of the J774 cells. One hundred of the macrophage supernatants were obtained 72 h after adding the CuNPs to the culture medium. Griess reagents A and B (60 μL from each) were poured into a 96-well culture plate. Finally, NO production was measured by reading the plates at 540 nm in an ELISA reader (BioTek-ELX800) [16].

### 2.6. Cytotoxic Effects of CuNPs on J774-A1 Cells

Here, the cytotoxic activity of CuNPs on J774-A1 cells was assessed by incubating the cells (5 × 10^5^) with different concentrations of CuNPs (0 to 100 mg/mL) at 37 °C in 5% CO_2_ for 2 days in 96-well plates. Finally, J774-A1 cell viability was evaluated by means of the MTT ([3-(4,5-dimethylthiazol-2-yl)-2,5-diphenyl tetrazolium bromide)]) assay, and the results are presented as the percentage of dead cells in comparison to J774-A1 cells with no drug (100% of viability). The 50% cytotoxic concentrations (CC_50_ values) were measured by the Probit test in SPSS software [17]. Moreover, to assess the toxicity and activity of CuNPs, the selectivity index (SI) was measured according to the CC_50_ calculation for J774-A1 cells/IC_50_ for *L. major* amastigote forms [17].

### 2.7. In Vivo Experiments

#### 2.7.1. Ethical Statement

This study was reviewed and approved by the Ethics Committee of the Department of Biological Sciences at Shaqra University, Saudi Arabia (No. SH17-2020).

#### 2.7.2. Inducing Cutaneous Leishmaniasis in BALB/c Mice

**Figure 1:**
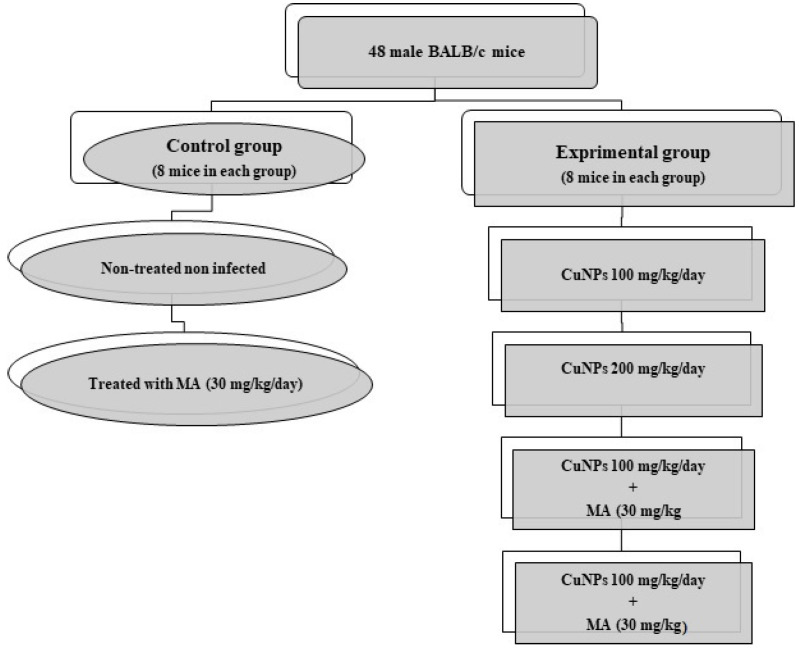
The experimental design of the present study.

Forty-eight male BALB/c mice, 6–8 weeks old, were randomly divided into six groups (8 mice per group) and kept in a colony room with a 12-h/2-h light/dark cycle at 21 ± 2 °C (Figure 1). For inducing CL in mice, 100 µL of promastigotes of *L. major* (2 × 10^6^ cells/mL) in the stationary phase was inoculated subcutaneously at the base of the tail [16].

#### 2.7.3. Treating Infected Mice

In the 6th week, when CL lesions appeared, treatment was initiated. Then, various concentrations of CuNPs (100 and 200 mg/kg, as an ointment), alone or combined with MA (30 mg/kg), were used topically for each tested group once a day for 4 weeks. By means of a Vernier caliper, the diameter of the CL lesions was calculated before, as well as after, the treatment with CuNPs. Animals in the control group received equivalent volumes of the vehicle as treatment animals (saline). The positive control group received MA (30 mg/kg) as the intralesional injection. In addition, the mean number of parasites (parasite load) was measured through impression smears obtained from the lesions. After fixing the smears with methanol and, then staining them with Giemsa, they were examined with a light microscope to calculate the parasite load in each tested group [16].

### 2.8. Statistical Analysis

All of the experiments were carried out in triplicate. The SPSS statistical package, version 22.0 (SPSS, Inc., Chicago, IL, USA), was applied for data analysis. Here, the alterations in the test and control groups were examined by a *t*-test. Furthermore, *p* < 0.05 was considered statistically significant.

## 3. Results

### 3.1. Characterization of Green Synthesized Cu NPs

**Figure 2:**
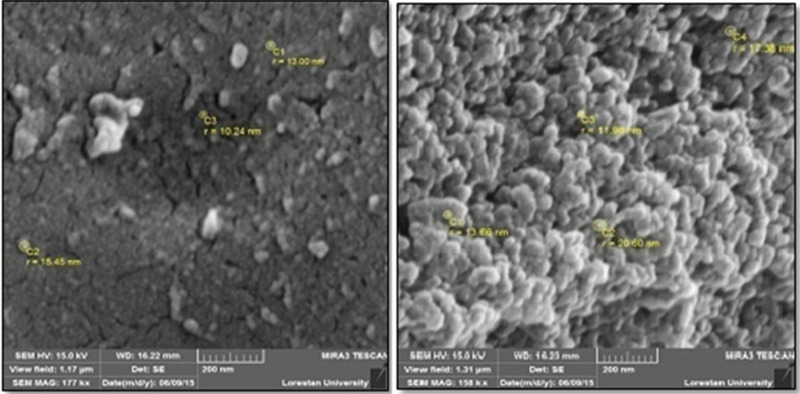
Scanning electron microscope of copper nanoparticles synthesized using methanolic extract of *Capparis spinosa* fruit.

As shown in Figure 2, the SEM image of green synthesized CuNPs demonstrated that these NPs have a spherical shape, whereas the particle size was observed to be 17 to 41 nm.

### 3.2. In Vitro Antiamastigote Effects

**Figure 3:**
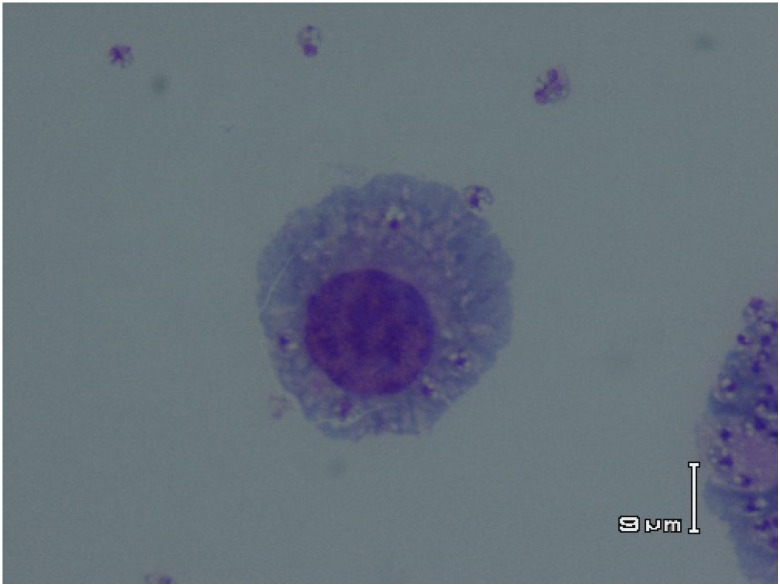
The J774-A1 macrophage cells infected with *L. major* amastigotes after treatment with various concentrations of copper nanoparticles (CuNPs).

The obtained findings show that CuNPs, especially combined with MA, significantly (*p* < 0.001) suppressed the growth rate of *L. major* amastigotes, based on a dose-dependent mode. The obtained IC_50_ values were 116.8 ± 3.05, 52.6 ± 2.15, and 21.3 ± 0.42 μg/mL for the CuNPs alone, MA alone, and CuNPs along with MA, respectively (Figure 3, Table 1).

### 3.3. Inhibiting Infection in Macrophage Cells

The obtained findings revealed that the nontreated *L. major* promastigotes were able to infect 81.3% of the J774-A1 cells, whereas promastigotes that were preincubated in CuNPs at concentrations of 10 and 20 µg/mL had the ability to infect only 39.3% and 21.4% of the J774-A1 macrophage cells; in addition, preincubation with CuNPs at 10 µg/mL + MA (10 µg/mL) and CuNPs 20 µg/mL + MA (10 µg/mL) caused 88.6 and 93.1% infectiveness reduction in promastigotes (Table 2).

### 3.4. Nitric Oxide Production

The results demonstrated that the CuNPs significantly (*p* < 0.05) triggered the production of NO in a dose-dependent manner. Table 3 shows the production of NO by CuNPs at concentrations of 10, 20, and 30 µg/mL, compared to the nontreated J774-A1 cells.

### 3.5. Cytotoxic Effects of CuNPs on J774-A1 Cells

The obtained results of MTT assay show that CuNPs had no significant cytotoxicity in J774 cells. The CC_50_ value of the CuNPs was 1325.4 ± 8.15 μg/mL. The SI of greater than 10 for CuNPs indicated its safety to the J774-A1 cells and specificity to the parasite (Table 1).

### 3.6. Effect of CuNPs on the Induced CL in BALB/c Mice

**Figure 4:**
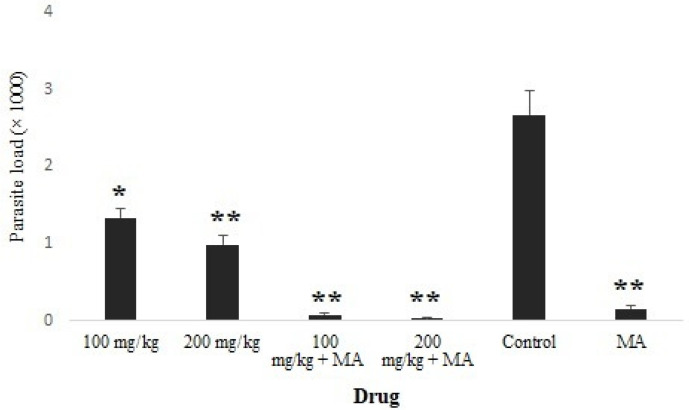
Comparison of the mean number of parasites (parasite load) in infected mice after treatment with various concentrations of CuNPs alone and along with MA, compared with the control group. * *p* < 0.05; ** *p* < 0.001.

**Figure 5:**
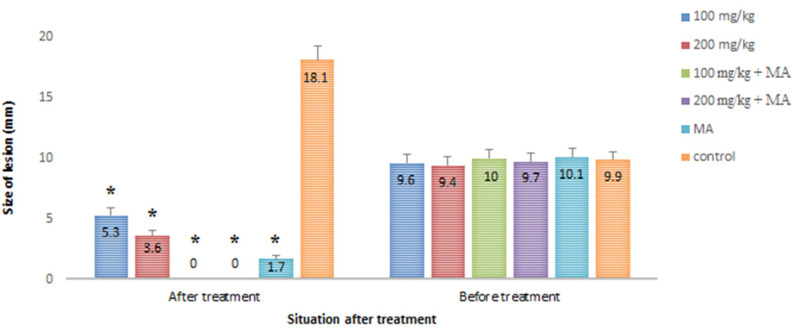
Antileishmanial effects of CuNPs alone and along with MA on the size of lesions in BALB/c mice infected by *L. major*. * *p* < 0.001.

The obtained results showed that the mean number of parasites was significantly (*p* < 0.05) reduced in the infected mice treated with CuNPs, especially among infected mice treated with CuNPs 100 and 200 mg/kg combined with MA (Figure 4). However, there was no significant difference in the mean number of parasites between 100 mg/kg CuNPs + MA and 200 mg/kg CuNPs + MA. Figure 5 shows the mean diameter of lesion size in the infected mice after 30 days of treatment in comparison with the control group. Complete recovery (100%) was observed among the infected mice treated with CuNPs 100 and 200 mg/kg combined with MA. The mean diameter of the lesions decreased by 43 and 58 mm after treating the groups with concentrations of 100 and 200 mg/mL CuNPs, respectively. However, in the untreated mice, the mean diameter of the lesions increased by 8.2 mm.

## 4. Discussion

Since there is no effective vaccine for preventing cutaneous leishmaniasis and drug resistance has been reported in some *Leishmania* spp. in recent years, improving and upgrading the existing treatment drugs and also discovering new and alternative agents are the best strategies for controlling and managing this disease [5,6]. In recent years, using nanoparticles for antimicrobial goals has been of great interest to many researchers around the world [18]. Currently, different physical and chemical methods have been considered to synthesize nanoparticles with certain size and lower toxicity. Among these procedures, green synthesis is described as one of the most standard, reliable, supportable, and eco-favorable synthesis approaches for synthesizing applicable and safe nanoparticles [10]. Nowadays, the green synthesis of metal nanoparticles by means of herbal products is considered to be a favored choice, rather than bacteria- and/or fungi-mediated synthesis. Due to its ease of synthesis, facility, biocompatibility, and environmentally friendliness in nature, green synthesis has been broadly used against microbial pathogens, alone and combined with the existing, conventional antimicrobial drugs. The present study aims to evaluate the antileishmanial effects of CuNP green synthesized by *C. spinosa* fruit extract alone and combined with MA in vitro and in vivo.

The obtained IC_50_ values showed that CuNPs, especially combined with MA, significantly (*p* < 0.001) inhibited the growth rate of the intracellular amastigote forms of *L. major* in a dose-dependent manner. The results of the in vivo assay demonstrated that the mean number of parasites was significantly (*p* < 0.05) reduced in the infected mice that were treated with CuNPs, especially combined with MA in a dose-dependent response. However, complete recovery (100%) was observed among the infected mice treated with CuNPs 100 and 200 mg/kg combined with MA. The mean diameter of the lesions decreased by 43 and 58 mm after treating the groups with concentrations of 100 and 200 mg/mL of CuNPs, respectively. In the untreated mice, the mean diameter of the lesions increased by 8.2 mm. Recently, Alizadeh et al. (2019) demonstrated that CuNPs at a concentration of 1 μM enhanced wound healing in the shortest time by developing granulation tissue and increasing vascularity [19]. Tiwari et al. (2013) showed the potent wound healing activity of biosynthesized CuNPs gel (0.1%) by reducing wound size by 92% in Wistar albino rats [20].

Considering the antiparasitic effects of CuNPs, in the study conducted by Malekifard et al. (2020), it was proven that copper oxide nanoparticles at a concentration of 600 µg/mL significantly reduced the viability of *Giardia lamblia* cysts after 3 h of treatment [21]. Previous studies also showed the potent antiparasitic effects against some protozoan parasites such as *Entamoeba histolytica* and *Cryptosporidum parvum* with IC_50_ values of 0.13 and 0.72 mg/L, respectively [22]. In addition, the antibacterial effects of CuNPs have been reported against a wide range of Gram-positive (e.g., *Listeria monocytogenes*, *Staphylococcus aureus*) and Gram-negative (e.g., *Salmonella enteric, Campylobacter jejuni, Escherichia coli*) bacteria, as well as some pathogenic fungi strains (e.g., *Aspergillus niger*) [23,24,25].

Even though the precise antimicrobial action mechanisms of CuNPs are not yet agreed, Mahmoodi et al., have recently reported that copper, through affecting the sulfhydryl groups, destroys proteins in the bacteria structure [24]. Moreover, Chatterjee et al. demonstrated that CuNPs with different mechanisms, such as degradation of bacterial DNA, lipid peroxidation, and production of reactive oxygen, cause cell wall destruction and subsequent bacteria death [26].

Based on the previous studies, infectivity or inhibition of infection in macrophage cells is considered to be one of the main pathogenic and biological factors of *Leishmania* parasites [16]. The obtained findings demonstrated that the nontreated *L. major* promastigotes were able to infect 81.3% of the J774-A1 cells, whereas promastigotes that were preincubated in CuNPs at concentrations of 10 and 20 µg/mL had the ability to infect only 46.3% and 21.4% of the J774-A1 macrophage cells. Today, NO is considered to be one of the main mediators of immunity produced in macrophages, which plays a critical role in the control of *Leishmania* parasites [27]. Here, we assessed NO production in macrophages treated with different concentrations of CuNPs. The obtained results showed that CuNPs at concentrations of 10 and 20 µg/mL significantly (*p* < 0.05) triggered the production of NO in a dose-dependent manner compared to the nontreated the J774-A1 cells.

Considering the cytotoxicity of CuNPs, the obtained results of the MTT assay showed that CuNPs had no significant cytotoxicity in J774 cells. The CC_50_ value of the CuNPs was 1325.4 ± 8.15 μg/mL. The SI of greater than 10 for CuNPs indicated its safety to the J774-A1 cells and specificity to the parasite. In line with our results, Prasad et al. (2017) demonstrated that CuNPs had no cytotoxicity at doses of 0.5 to 1.5 μM on prostate cancer (PC-3) cell lines [28]. In addition, Ostaszewska et al. (2018) also showed that CuNPs at a concentration of 0.15 mg/mL had no significant cytotoxicity on rainbow trout (*Oncorhynchus mykiss*) hepatocytes after 4 weeks of incubation time [29]. Thus, it can be suggested that CuNPs had no cytotoxicity for mammalian cells.

Considering the in vivo toxicity of CuNPs, the results demonstrated the high efficacy of CuNPs at doses of 100 and 200 mg/kg, especially in combination with MA for CL treatment in mice. Recently Khatami et al. (2020) have demonstrated that oral administrations of green synthesized CuNPs at doses of 1000, 2000, and 5000 μg/kg had no toxicity on the liver functions and hematological parameters of mice [30]. In another study conducted by the authors of the present study, there was no significant effect on the sensory-motor test in mice after the oral administration of CuNPs at doses of 25, 50, and 75 mg/kg [31]. These studies indicate the few side effects and low toxicity of CuNPs, but since, in this study, we used CuNPs topically, further studies are required to elucidate the histological and toxicological issues.

Toxicity of these nanoparticles seems necessary.

## 5. Conclusions

The findings of the present study demonstrated high potency and synergistic effect of CuNPs alone and combined with MA in inhibiting the growth of amastigote forms of *L. major,* as well as recovering and improving CL induced by *L. major* in BALB/c mice. The findings also indicated that, although the possible antileishmanial mechanisms of CuNPs were not obviously understood, triggering NO was one of the main antileishmanial mechanisms of CuNPs. Additional supplementary studies, especially in clinical settings, are required in this regard.

## Figures and Tables

**Table 1:** The IC_50_ and CC_50_ values (µg/mL) determined for the CuNPs alone and along with meglumine antimoniate (MA), compared with the MA alone, and their selectivity index (SI) against intramacrophage amastigote forms of *Leishmania major*.

Tested Material	IC_50_ (µg/mL) for *L. major* Amastigote	CC_50_ (µg/mL) of the J774-A1 Cells	SI
CuNPs	116.8 ± 3.05	1325.4 ± 8.15	11.34
MA	52.6 ± 2.15	1125.6 ± 11.60	21.39
CuNPs + MA	21.3 ± 0.42	396.3 ± 8.51	18.60

**Table 2:** Inhibition of the infection in macrophage cells after treatment of *L. major* promastigotes with CuNPs. Data are expressed as the mean ± SD (n = 3).

Promastigotes	Percentage of Infected Macrophages	Infectiveness Reduction (%)
Nontreated	81.3 ± 3.15	-
CuNPs (10 µg/mL)	39.3 ± 2.33	42.0
CuNPs (20 µg/mL)	21.4 ± 1.51	59.9
CuNPs (10 µg/mL) + MA (10 µg/mL)	9.3 ± 0.61	88.6
CuNPs (20 µg/mL) + MA (10 µg/mL)	5.6 ± 0.15	93.1

**Table 3:** Comparison of NO production in J774-A1 macrophage cells after treatment with various concentrations of CuNPs.

Concentration (µg/mL)	Production of Nitric Oxide (nM)
10	8.3 ± 0.55
20	9.6 ± 0.74
30	17 ± 1.55
Nontreated	10.6 ± 1.15

## Data Availability

All data generated or analyzed during this study are included in this published article.
